# YopP-Expressing Variant of *Y. pestis* Activates a Potent Innate Immune Response Affording Cross-Protection against Yersiniosis and Tularemia

**DOI:** 10.1371/journal.pone.0083560

**Published:** 2013-12-16

**Authors:** Ayelet Zauberman, Yehuda Flashner, Yinon Levy, Yaron Vagima, Avital Tidhar, Ofer Cohen, Erez Bar-Haim, David Gur, Moshe Aftalion, Gideon Halperin, Avigdor Shafferman, Emanuelle Mamroud

**Affiliations:** Department of Biochemistry and Molecular Genetics, Israel Institute for Biological Research, Ness-Ziona, Israel; University of Louisville, United States of America

## Abstract

Plague, initiated by *Yersinia pestis* infection, is a rapidly progressing disease with a high mortality rate if not quickly treated. The existence of antibiotic-resistant *Y. pestis* strains emphasizes the need for the development of novel countermeasures against plague. We previously reported the generation of a recombinant *Y. pestis* strain (Kim53ΔJ+P) that over-expresses *Y*. *enterocolitica* YopP. When this strain was administered subcutaneously to mice, it elicited a fast and effective protective immune response in models of bubonic, pneumonic and septicemic plague. In the present study, we further characterized the immune response induced by the Kim53ΔJ+P recombinant strain. Using a panel of mouse strains defective in specific immune functions, we observed the induction of a prompt protective innate immune response that was interferon-γ dependent. Moreover, inoculation of mice with *Y. pestis* Kim53ΔJ+P elicited a rapid protective response against secondary infection by other bacterial pathogens, including the enteropathogen *Y*. *enterocolitica* and the respiratory pathogen *Francisella tularensis.* Thus, the development of new therapies to enhance the innate immune response may provide an initial critical delay in disease progression following the exposure to highly virulent bacterial pathogens, extending the time window for successful treatment.

## Introduction

Plague is a fatal disease that has caused millions of deaths in three world pandemics, and it remains a public health concern in some regions of the world [1] [2]. The disease, initiated by infection with the bacterial pathogen *Yersinia pestis*, is manifested in two main forms: pneumonic plague and bubonic plague. Primary pneumonic plague is the more fatal form of the disease, although it is less frequent in nature; it results from the inhalation of *Y. pestis*-containing droplets or aerosols. Bubonic plague develops following transmission of the pathogen from rodent reservoirs to humans by infected fleas [1]. Bacteria migrate through the cutaneous lymphatics to regional lymph nodes, where they multiply rapidly, followed by colonization of the spleen and liver and, finally, bacteremia and systemic dissemination to other tissues. Nonspecific symptoms start several days after infection and are followed by the development of swollen lymph nodes, which are termed buboes [3].

The medical treatment of plague should be initiated as early as possible to be effective. Antibiotic administration is the well-established first-line therapy against plague. However, the existence of antibiotic-resistant *Y. pestis* strains highlights the need for the development of new therapeutic tools [4]. Three main strategies have been adopted to achieve this goal: a. Search for anti-infective agents that can inhibit the activity of specific essential virulence systems or virulence factors. These agents include small molecules that target the type III secretion system (T3SS) apparatus and specific T3SS effectors [5]–[8]. b. Development of subunit vaccine candidates that extend the spectrum of the immune response generated by the F1/LcrV-based vaccines are also promising [9], [10]. c. Attenuated *Y. pestis* strains have been used in an effort to reveal potential targets for new therapeutic measures or to elucidate elements of the host immune system that are required for the generation of protective immunity against plague [11]–[16]

We have recently shown that a newly engineered strain of *Y. pestis*, Kim53ΔJ+P, is dramatically attenuated in a mouse model of bubonic plague (LD_50_>10^7^ cfu), whereas its virulence is retained in mouse models of pneumonic and systemic plague [17]. This strain was generated by the over-expression of the *Y. enterocolitica* 0:8 T3SS effector YopP in the virulent yopJ-deleted Kimberley53 *Y. pestis* strain. YopP is homologous to the endogenous *Y. pestis* YopJ; both are known to inhibit the pro-inflammatory response in target host cells [18]–[20]. However, the recombinant YopP-expressing strain was found to induce cell death more effectively than the wild-type strain, most likely due to differences in YopJ/YopP translocation efficiency and allelic variations among the genes encoding these effectors [17], [21]. These results are similar to the findings of Brodsky *et al*., who demonstrated that the ectopic expression of YopP from *Y. enterocolitica* in *Y. pseudotuberculosis* led to attenuation following the oral infection of mice [22]. Interestingly, the *Y. pestis* Kim53ΔJ+P strain induced a rapid and effective systemic immune response that provided protection against subcutaneous (s.c.), intranasal (i.n.) and intravenous challenges with a fully virulent *Y. pestis* strain [17].

In the present study, we further characterized the rapid immune response induced by the Kim53ΔJ+P recombinant *Y. pestis* strain in an effort to uncover a potential new avenue for the development of protective measures against plague. Using immunodeficient mouse strains, we observed the induction of a prompt but transient protective innate immune response that was interferon-γ dependent. This response appeared to postpone disease progression and allow the development of adaptive immunity. Consistent with the relatively low specificity characterizing the rapid induction of the innate response, Kim53ΔJ+P-infected wild-type mice were also protected from a lethal challenge with the enteropathogen *Y. enterocolitica* and furthermore with a non-related respiratory pathogen, *Francisella tularensis* subspecies holarctica LVS.

## Materials and Methods

### Bacterial strains

The bacterial strains used in this study are listed in Table 1. *Yersinia* strains were routinely grown on brain heart infusion agar (BHIA, Difco) for 48 hours at 28°C. Ampicillin-resistant *Y. pestis* strains (Kim53pGFP, Kim53ΔJ+P) were grown on BHIA supplemented with 100 µg/ml ampicillin (Sigma, Israel). Live *F. tularensis* vaccine strain (ATCC 29684) stocks were plated on GCHI agar (GC Medium base [Difco Laboratories] supplemented with 1% hemoglobin and 1% Iso-VitaleX [BD, France]).

**Table 1:** Bacterial strains used in this study.

Strains	Relevant characteristics	Reference
***Yersinia pestis***		
Kimberley53 (Kim53)	Fully virulent *Y. pestis* strain (biovar Orientalis)	[11], [49]
Kim53pGFP	Kim53 carrying pGFPuv (Clontech)	[50]
Kim53Δ*yop*J+p*yop*P	*yop*J-deleted Kim53 over-expressing YopP of *Y. enterocolitica* WA 0:8	[17]
Kim53ΔpCD1ΔpPCP1	Spontaneously pPCP1 and pCD1-cured Kim53	[17]
EV76	*pgm*- (Girard's strain)	[49]
***Yersinia enterocolitica***		
WA O:8	Virulent *Y. enterocolitica* strain	[51]
***Francisella tularensis***		
Holarctica LVS	Live vaccine strain (ATCC 29684)	[52]

### Ethics statement

This study was carried out in strict accordance with the recommendation in the Guide for the Care and Use of Laboratory Animals of the National Institute of Health. All animal experiments were performed in accordance with Israeli law and were approved by the Ethics Committee for animal experiments at the Israel Institute for Biological Research. (Permit Number: IACUC-IIBR M-32-2-09, IACUC-IIBR M-57-09, IACUC-IIBR M-58-11 and IACUC-IIBR M-62-11, IACUC-IIBR M-23-12, IACUC-IIBR M-26-13). During the experiments mice were monitored daily. Humane endpoints were used in our survival studies. Mice exhibiting loss of the righting reflex were euthanized by cervical dislocation. Analgesics were not used as they may have affected the experimental outcomes of the studies.

### Animals

Five- to six-week-old female OF1 mice were purchased from Charles River Laboratories (IFFA CREDO S.A., France). Female C67BL/6J, µMT (B6.129S2-Ighm^tm1Cgn^/J), RAG1^-/-^ (B6;129S7-Rag1^tmMom^/J), Kit*^wsh^* ^-/-^ (B6.CgKitWsh/HNih rLae BsmJ), IFNγ^-/-^ (B6.129S7-*Ifng* ^tm1Ts^/J), TCRbd^-/-^ (B6.129P2-Tcrb^tm1Mom^Tcrd^tm1Mom^/J), and CD4^-/-^ (B6.129S2-*Cd4^tm1Mak^*/J) mice (6 to 8 weeks old) were purchased from Jackson Laboratories (ME, USA) and randomly assigned into cages in groups of 6 animals. The mice were allowed free access to water and rodent diet (Harlan, Israel).

### Animal studies

*Yersinia* strains were grown on BHIA plates (Difco) for 48 hours at 28°C. Several colonies were suspended in a sterile saline solution, diluted to the desired infectious dose and used for the challenge. Mice (6/group, unless stated otherwise), were injected with 0.1 ml of suspension containing the indicated amount of bacterial cells s.c. in their lower or upper back or intraperitoneally (i.p.). Bacterial cell counts were performed by serial dilution plating on BHIA. Unless otherwise indicated, the challenged animals were monitored daily for 35 days. The *F. tularensis* LVS strain was grown at 37°C to the mid-log phase (optical density of 0.1–0.2 at 660 nm) in TSBC (TSB [Difco] supplemented with 0.1% cysteine). The bacteria were washed and then re-suspended at the desired concentration in PBS. The bacteria were instilled i.n. (25 µl) to ketamine/xylazine-anesthetized mice or injected i.p. (0.5 ml). Experiments evaluating the ability of *Y. pestis* Kim53ΔJ+P to protect against *Y. enterocolitica* and *F. tularensis* were performed with OF1 outbred mice. Results from animal studies were obtained from at least two repeats.

### Antibody titer analysis

Titers of anti-*F. tularensis* antibodies in serum samples were determined by ELISA in 96-well microtiter plates coated with 100 µl of 10^8^ CFU/ml formalin-inactivated LVS. Antibody titers were expressed as reciprocal geometric mean titers (GMTs). The limit of detection for this assay was the reciprocal GMT value of 40.

### Statistical analyses

GraphPad statistical software was used for the statistical analyses. Survival curves were compared using the log-rank test. In all analyses, P values equal to 0.05 served as the limit of significance.

## Results and Discussion

### *Y. pestis* over-expressing YopP activates a transient innate protective response

In mice, the s.c. administration of Kim53ΔJ+P induces a highly rapid systemic resistance against *Y. pestis* strains [17]. To characterize the contribution of innate immunity to the Kim53ΔJ+P-activated protective response, we used immunodeficient RAG1^-/-^ mice, which lack the capacity to mount an adaptive immune response.

**Figure 1:**
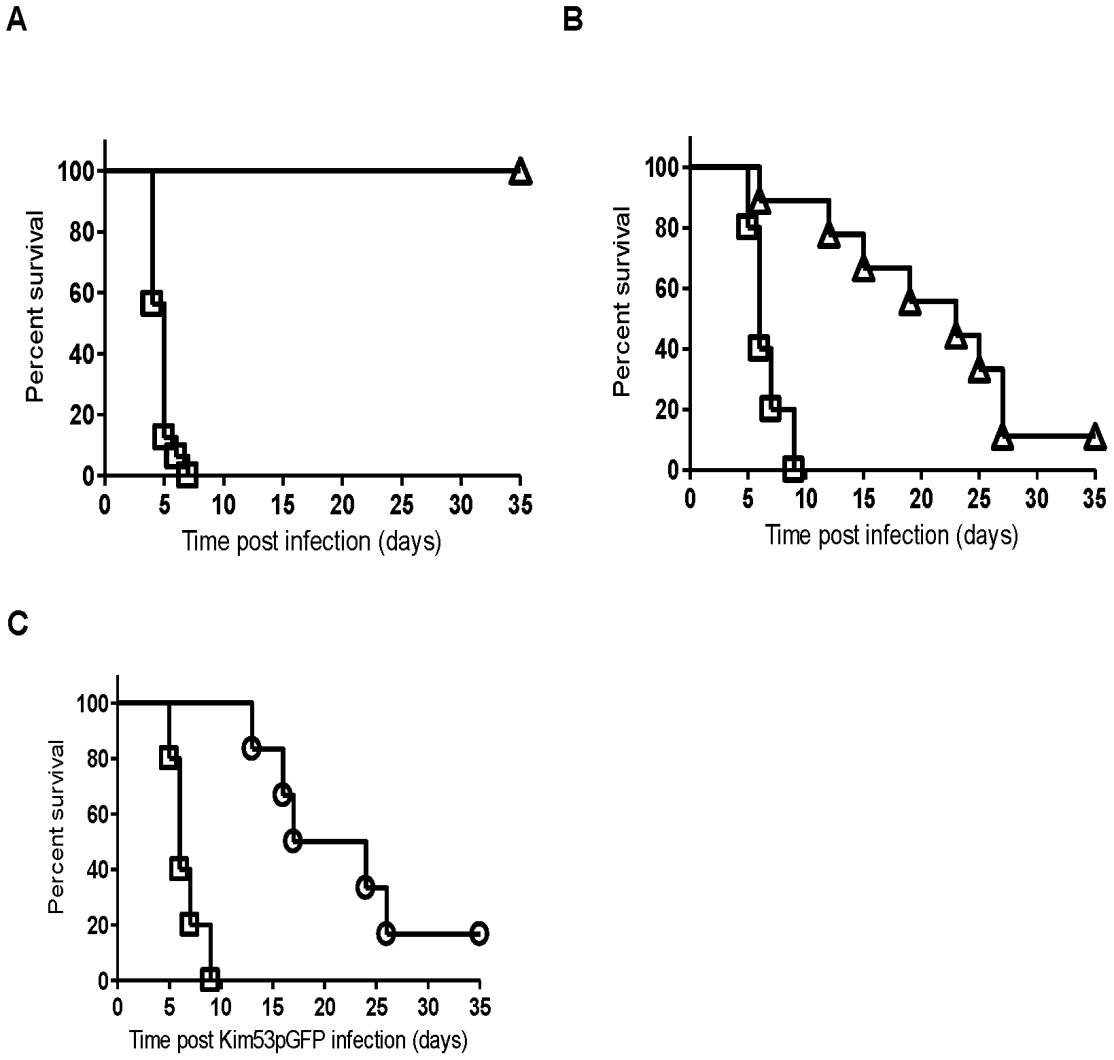
Transient protective immunity induced by Kim53ΔJ+P in immunodeficient RAG1^-/-^ mice. C57BL/6J mice (A) and their isogenic RAG1^-/-^ mice (B) were infected s.c. with either 100 cfu of Kim53pGFP strain (square, five mice/group) or 1×10^4^ cfu of Kim53ΔJ+P (triangle, nine mice/group). (C) RAG1^-/-^ mice were either only infected s.c. with 100 cfu of Kim53pGFP (square) or infected with 1×10^4^ cfu of Kim53ΔJ+P and challenged s.c. with Kim53pGFP three days later (circle, six mice/group).

Parental C57BL/6J mice and their isogenic RAG1^-/-^ mice were infected s.c. with Kim53ΔJ+P (10^4^ cfu) or with the fully virulent *Y. pestis* strain Kim53pGFP (10^2^ cfu) as a control. As expected, all wild-type C57BL/6J mice survived the Kim53ΔJ+P infection, whereas those infected with Kim53pGFP succumbed to the disease within seven days (Figure 1A). All immunodeficient RAG1^-/-^ mice infected with Kim53pGFP succumbed to the disease within nine days, whereas high survival rates of 100-89% were observed for Kim53ΔJ+P-infected RAG1^-/-^ mice during the initial nine days post-infection (Figure 1B). This result suggests that following the s.c. infection of RAG1^-/-^ mice with Kim53ΔJ+P, a rapid protective innate immune response was evoked. However, the protective response was transient as the survival rate declined gradually to 14% by day 35 post-infection (mean time to death of 19 days in the Kim53ΔJ+P-infected mice compared with 6.6 days in the RAG1^-/-^ mice infected with Kim53pGFP).

In a subsequent cross-protection experiment, we assessed the ability of the innate immune response induced by Kim53ΔJ+P to provide rapid systemic protection against the challenge with a fully virulent *Y. pestis* strain inoculated s.c. at a different remote site. RAG1^-/-^ mice were infected s.c. with 10^4^ cfu of Kim53ΔJ+P and were challenged s.c. with a lethal dose of 10^2^ cfu of the virulent Kim53pGFP strain three days later. Indeed, the Kim53ΔJ+P-induced protective response in the RAG1^-/-^ mice was also effective in postponing the time to death following Kim53pGFP infection (Figure 1C). Importantly, the survival curve of co-infected mice was statistically indistinguishable from that of mice infected with Kim53ΔJ+P alone (P = 0.97 by the log-rank test, Figures 1B and 1C). These results indicate that a potent protective innate response can delay mouse mortality following infection with a highly virulent *Y. pestis* strain and even cure a small but distinct proportion of *Y. pestis*-infected mice. One may speculate that in wild-type mice this initial delay in disease progression provides the host with the time needed to develop an effective adaptive response against *Y. pestis* infection.

In an effort to characterize the distinct innate immune response induced rapidly following infection with Kim53ΔJ+P, we compared the levels of various classes of innate immune cells in the draining inguinal lymph node (ILN) and spleen at 24 and 48 hours post-infection with Kim53ΔJ+P and the virulent Kim53pGFP strain using flow cytometry. However, no significant differences in the levels of neutrophils, macrophages and dendritic cells were observed between mice infected with Kim53ΔJ+P and Kim53pGFP (data not shown). Mast cells (MCs) are another type of pro-inflammatory cell located throughout the skin, and they are considered to be among the first cells encountered by invading pathogens. MCs can induce rapid innate immune responses through the release of mediators [23]–[25]. Interestingly, histamine, which is one of these MC mediators, has been shown to be important for controlling *Y. enterocolitica* infection in mice [26]. These mast cell features led us to evaluate their involvement in the specific immunity induced by s.c. infection with Kim53ΔJ+P. However, the infection of MC-deficient mice (Kit*^wsh^* ^-/-^) with 10^4^ cfu of Kim53ΔJ+P via the s.c. route resulted in 100% survival. Furthermore, all Kim53ΔJ+P–infected Kit*^wsh^* ^-/-^ mice were protected against the challenge with a lethal dose of the virulent Kim53pGFP strain, which was administered s.c. three days later. These results indicate that MC activation is dispensable for the rapid induction of protective immunity by Kim53ΔJ+P.

### Interferon-gamma is essential for the rapid induction of the Kim53ΔJ+P-mediated protective response

**Figure 2:**
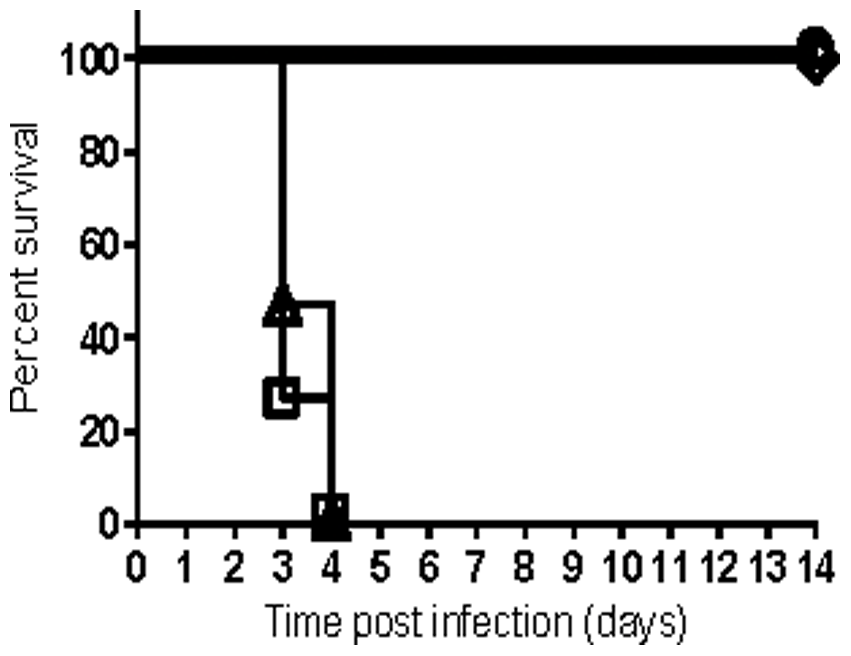
IFNγ is essential for the Kim53ΔJ+P-mediated rapid protective response. IFNγ-deficient mice (IFNγ^-/-^) were infected with 1×10^4^ cfu of the following strains: Kim53ΔJ+P (triangle, twelve mice/group), Kim53pGFP (square, four mice/group), EV76 (circle, four mice/group) and Kim53ΔpCD1ΔpPCP1 (diamond, four mice/group).

Interferon-gamma (IFNγ) is a pleiotropic cytokine that modulates the development of both the innate and adaptive immune responses [27]. Based on the key role that IFNγ plays in the immune system and its previously demonstrated role in the defense against *Yersinia* species [28]–[35], we evaluated the contribution of IFNγ to Kim53ΔJ+P-mediated immunity. All mice infected s.c. with 10^4^ cfu of Kim53ΔJ+P succumbed to the disease within four days, which was similar to mice infected with the fully virulent *Y. pestis* strain (Figure 2). Restoration of the virulent phenotype was unique to Kim53ΔJ+P, as IFNγ-deficient mice infected with other attenuated *Y. pestis* strains, such as the vaccine strain EV76 or the pCD1^−^/pPCP1^−^ derivative of Kim53, did not develop any disease symptoms (Figure 2). These observations clearly indicate that IFNγ is a key element of Kim53ΔJ+P-mediated immunity. Moreover, the rapid kinetics of disease progression and mortality of all Kim53ΔJ+P-infected IFNγ^-/-^ mice within four days post-infection suggests that IFNγ is important for the induction of protective innate responses by Kim53ΔJ+P prior to the development of adaptive immunity.

**Figure 3:**
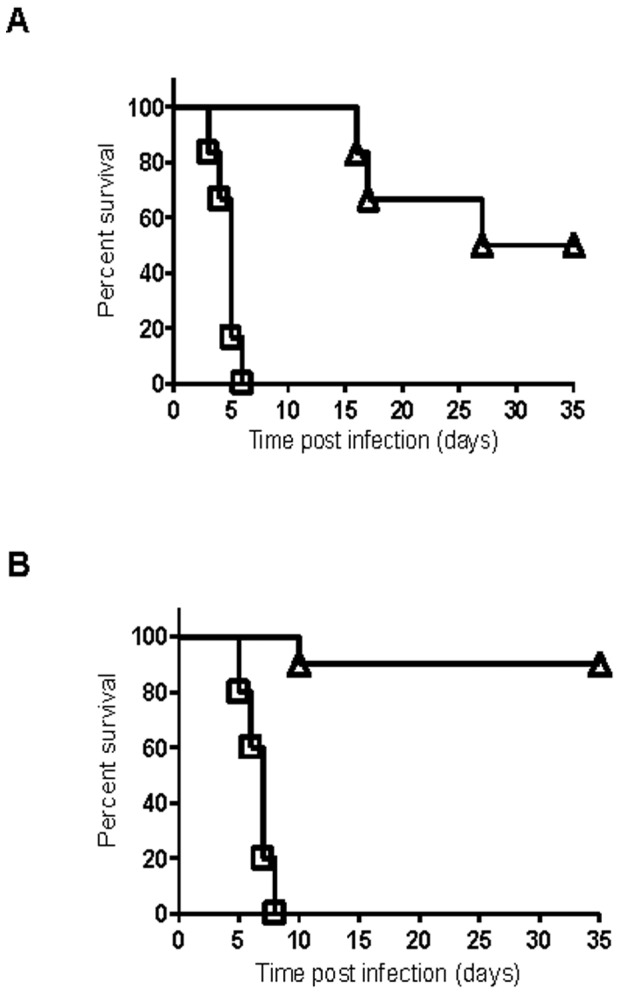
Contributions of T cells and B cells to the protective immunity induced by Kim53ΔJ+P. TCRbd^-/-^ mice lacking functional T cells (A) and µMT mice lacking functional B cells (B) were infected s.c. with 10^4^ cfu of Kim53ΔJ+P (triangle, ten mice/group) or 100 cfu of Kim53 (square, five mice/group). Survival was monitored for 35 days.

Numerous studies have demonstrated that IFNγ can activate innate immune cells to exert a potent antimicrobial effect against intracellular pathogens, including *Legionella pneumophila* [36], *F. tularensis* [37], [38] and *Mycobacterium tuberculosis* [39]. However, in the case of *Y. pestis*, the *ripA* gene located within the *pgm* locus was shown to contribute to pathogen survival in macrophages activated by IFNγ in vitro following infection [40]. Recent in vivo studies have shown that neutralization of tumor necrosis factor alpha (TNFα) and IFNγ increases the lethality and bacterial burden of *pgm* ^−^negative *Y. pestis* strain D27 in naïve mice infected i.n. [41]. In addition, these cytokines were found to play a role in the F1/LcrV-mediated protective response in various mouse models of plague and are proposed to be released from T cells primed following vaccination [31], [34], [41]. In the present study, we further extend the observations regarding the contribution of IFNγ to host defense against plague and demonstrate that this cytokine plays an essential role in the rapid development of protective immunity against s.c. infection with the *pgm-*positive Kim53ΔJ+P *Y. pestis* strain in naïve mice (Figure 2). The rapid time course of disease progression in Kim53ΔJ+P-infected IFNγ^-/-^ mice suggests that adaptive cellular immunity, which typically requires a longer duration to develop, is not involved in the IFNγ-mediated early protective response against Kim53ΔJ+P. To further address this assumption, TCRbd^-/-^ mice, which lack functional T cells, were infected s.c. with 10^4^ cfu of Kim53ΔJ+P, and survival was monitored for 35 days. All TCRbd^-/-^ mice survived for two weeks after s.c. infection with 10^4^ cfu of Kim53ΔJ+P, suggesting that T cells are not required for the IFNγ-mediated early protective response against Kim53ΔJ+P (Figure 3A). However, by day 35 post-infection, 50% of the TCRbd^-/-^-infected mice succumbed to the infection (Figure 3A), indicating that T cell activity is required to augment the early protective innate response against Kim53ΔJ+P and that this activity makes an important contribution to host defense against Kim53ΔJ+P infection. Notably, the ability of T cells to help B cells produce antibodies appears to play a minor role in the Kim53ΔJ+P-mediated defense response, as 90% of µMT B cell-deficient mice (Figure 3B) and all CD4 T cell-deficient mice survived for 35 days following s.c. infection with 10^4^ cfu of Kim53ΔJ+P.

Currently, the exact mechanism by which IFNγ is involved in the Kim53ΔJ+P-mediated early innate immune response is unclear, and requires further studies. It should be noted, however, that the levels of IFNγ measured in the spleens of mice during the first 72 hours after infection with Kim53ΔJ+P were similar to their levels in mice infected with the virulent Kim53pGFP strain [17], suggesting that IFNγ might act locally following s.c. infection with Kim53ΔJ+P. Another possibility is that the expression of IFNγ is necessary, but not sufficient for Kim53ΔJ+P-mediated rapid protective response, and that the ability of the host to overcome the infection depends on another, yet unknown, factor(s).

### Kim53ΔJ+P activates cross-protection against other bacterial pathogens

Non-specific protective responses against secondary infection with heterologous bacterial pathogens have previously been described in the pioneering studies of Mackaness and Henderson in the 1960s [42], [43], and in later publications by Killar [44] and Elkins [45]. Additional work by Elkin *et al*., also described rapid induction of non-specific, IFN-γ-mediated protection against *F. tularensis* infection using bacterial DNA [46]. The rapid activation of innate immunity by Kim53ΔJ+P raised the intriguing question of whether s.c. infection with Kim53ΔJ+P would also elicit heterologous protective immunity against other bacterial pathogens.

**Figure 4:**
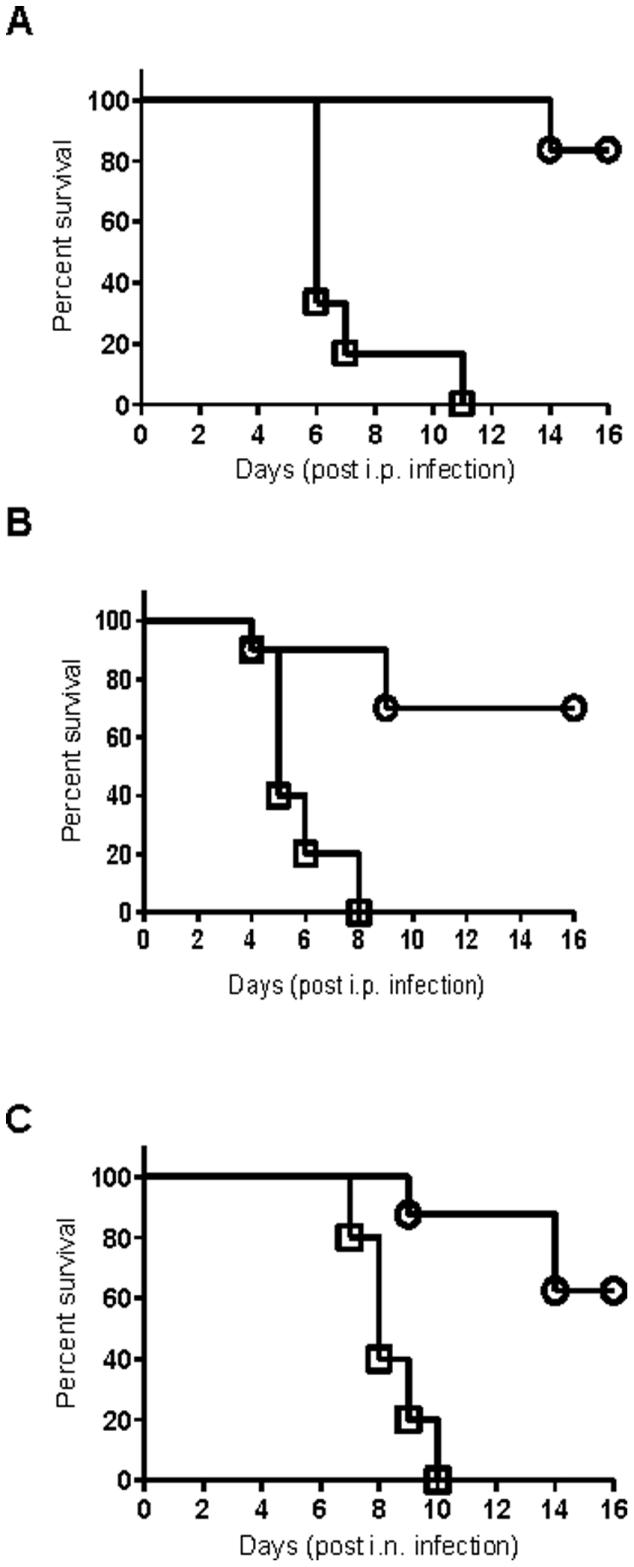
Kim53ΔJ+P induces cross-protection against *Y. enterocolitica* and *F. tularensis*. (A) Mice were either only infected i.p. with 1×10^3^ cfu of *Y. enterocolitica* WA (square, six mice/group) or infected s.c. with 1×10^4^ cfu of Kim53ΔJ+P and challenged i.p. with 1×10^3^ cfu of *Y. enterocolitica* WA three days later (circle, six mice/group). (B) Mice were either only infected either i.p. with 500 cfu of *F. tularensis* LVS (square, ten mice/group) or infected s.c. with 1×10^4^ cfu of Kim53ΔJ+P and challenged i.p. with 500 cfu of *F. tularensis* LVS three days later (circle, ten mice/group). (C) Mice were either only infected i.n. with 5000 cfu of *F. tularensis* LVS (square, eight mice/group) or infected s.c. with 1×10^4^ cfu of Kim53ΔJ+P and challenged i.n. with 5000 cfu of *F. tularensis* LVS three days later (circle, ten mice/group). Mortality was monitored for 16 days post-challenge.

We first evaluated whether Kim53ΔJ+P-mediated cross-protection could be induced against another member of the *Yersinia* genus, the food-born pathogen *Y. enterocolitica*, which causes gastroenteritis [47]. Mice were infected s.c. with 10^5^ cfu of Kim53ΔJ+P and were challenged i.p. three days later with a lethal dose of 2×10^3^ cfu of *Y. enterocolitica* WA O:8 strain. Although all mice infected with 2×10^3^ cfu of *Y. enterocolitica* alone succumbed to the disease within 11 days, a high survival rate of 80% was observed when the mice were challenged with *Y. enterocolitica* three days after infection with Kim53ΔJ+P (Figure 4A). This result indicated that the Kim53ΔJ+P-activated response can indeed provide rapid and effective cross-protection against another pathogen. However, because *Y. enterocolitica* and *Y. pestis* belong to the same genus and may share common antigenic determinants, we extended the evaluation of the protective immunity induced by Kim53ΔJ+P toward a non-related Gram-negative pathogen, *F. tularensis*, the etiologic agent of tularemia. Mice were infected i.p. and i.n. with a lethal dose of the *F. tularensis* subspecies holarctica LVS attenuated strain (500 cfu and 5,000 cfu, respectively). All infected mice exhibited disease symptoms (weight loss), and succumbed within ten days (Figures 4B and 4C). However, when the mice were first infected s.c. with Kim53ΔJ+P and challenged three days later with *F. tularensis* LVS, high survival rate of 70% was observed following the i.p. challenge and most notably, 63% survived the i.n. challenge (Figures 4B and 4C). In addition, substantial anti-LVS titers (10^3^–10^5^) were measured in the sera of mice surviving the infection with Kim53ΔJ+P and secondary challenge with *F. tularensis* LVS. These findings suggest that the initial non-specific immune response evoked by *Y. pestis* Kim53ΔJ+P delayed disease progression, allowing the development of a slower adaptive immune response against the *F. tularensis* LVS.

Notably, s.c. infection with the facultative intracellular pathogen *Y. pestis* Kim53ΔJ+P induced a prompt protective response against an unrelated intracellular pathogen that can replicate in the lung, spleen and liver. One can speculate that IFNγ, which was suggested in this study to be essential for the development of rapid protection against Kim53ΔJ+P, might also provide initial antigen-independent cross-protection against *F. tularensis* LVS due to its ability to activate macrophages. Indeed, it has been reported that rapid generation of a strong innate immune response mediated by macrophages and NK cells and dependent on IFNγ and TNFα, protects naïve mice against lethal infection with *F. tularensis* LVS [48].

Taken together, the present data indicate that the s.c. infection of mice with Kim53ΔJ+P activates an extremely rapid and potent protective response in which innate immunity appears to play a critical although transient role. IFNγ was found to be a key player in mediating this early, effectively induced protective response. Consistent with the relatively low specificity characterizing the rapid induction of the innate response, Kim53ΔJ+P-infected wild-type mice were also protected from a lethal challenge with other bacterial pathogens, including the enteropathogen *Y. enterocolitica* and the respiratory pathogen *F. tularensis* LVS. Thus, the development of new therapies to enhance the innate immune response may provide an initial critical delay in disease progression following the exposure to highly virulent bacterial pathogens such as *Y. pestis*, extending the time window for successful treatment.
