# Correction: Yopp-Expressing Variant of*Y. pestis* Activates a Potent Innate Immune Response Affording Cross-Protection against Yersiniosis and Tularemia

**DOI:** 10.1371/annotation/e6ae3cd6-3d55-4ec6-8437-19572280e260

**Published:** 2013-12-23

**Authors:** Ayelet Zauberman, Yehuda Flashner, Yinon Levy, Yaron Vagima, Avital Tidhar, Ofer Cohen, Erez Bar-Haim, David Gur, Moshe Aftalion, Gideon Halperin, Avigdor Shafferman, Emanuelle Mamroud

This article has been republished to correct an error in the title. 

