# Correction: YopP-Expressing Variant of *Y. pestis* Activates a Potent Innate Immune Response Affording Cross-Protection against Yersiniosis and Tularemia

**DOI:** 10.1371/annotation/0ec1f217-4e7a-4dee-9a71-1076a3ac73f1

**Published:** 2014-01-28

**Authors:** Ayelet Zauberman, Yehuda Flashner, Yinon Levy, Yaron Vagima, Avital Tidhar, Ofer Cohen, Erez Bar-Haim, David Gur, Moshe Aftalion, Gideon Halperin, Avigdor Shafferman, Emanuelle Mamroud

This article was republished on January 27th, 2014 to correct an error in the title. Please see the correct citation here:

Zauberman A, Flashner Y, Levy Y, Vagima Y, Tidhar A, et al. (2013) YopP-Expressing Variant of *Y. pestis* Activates a Potent Innate Immune Response Affording Cross-Protection against Yersiniosis and Tularemia. PLoS ONE 8(12): e83560. doi:10.1371/journal.pone.0083560

